# Bicuspid Aortic Valve in Pregnancy Complicated by Aortic Valve Vegetation, Aortic Root Abscess, and Aortic Insufficiency

**DOI:** 10.7759/cureus.20209

**Published:** 2021-12-06

**Authors:** Shan He, Christopher A Huynh, Yi Deng, Sandeep Markan, Anvinh Nguyen

**Affiliations:** 1 Anesthesiology, Baylor College of Medicine, Houston, USA

**Keywords:** infective endocarditis, aortic aneurysm, aortic valve insufficiency, congenital heart defects, bicuspid aortic valve disease

## Abstract

A 39-year-old patient presented to our Labor and Delivery unit with fever and nausea in the context of recent bacteriuria. She was found to be in sepsis due to an incidental bicuspid aortic valve (BAV) complicated by aortic valvular vegetations, severe aortic insufficiency, and aortic root abscess, requiring an emergent cesarean section. Three days after delivery, the patient successfully underwent a mechanical aortic valve replacement and root reconstruction. In this case report, medical, surgical, and anesthetic management of parturient patients with BAV are discussed. The management of this congenital valvulopathy and vasculopathy is complicated by the extensive hemodynamic and cardiovascular derangements that occur during pregnancy.

## Introduction

Congenital bicuspid aortic valve (BAV) is the most common congenital heart malformation [1,2]. It is frequently associated with valvular complications, including aortic regurgitation, aortic stenosis (AS), and infective endocarditis, and vascular complications, including aortic dilatation, dissection, and aneurysm [2-5]. As more patients with congenital heart disease thrive in adulthood and consider pregnancy, anesthesia providers must be familiar with the pathophysiology and management of BAV. This article was previously presented as a poster at the 2021 American Society of Anesthesiologists (ASA) Anesthesiology Annual Meeting on October 10, 2021.

## Case presentation

A 39-year-old G5P2022 female at 34 weeks of pregnancy presented to the hospital for evaluation of a one-week history of fever, chest pain, nausea, and vomiting. Her pregnancy had been complicated by two prior hospital visits for similar symptoms for Group B streptococcal (GBS) bacteriuria. The patient did not have a history of intravenous drug abuse.

On admission to the Labor and Delivery Unit, her vitals were notable for maternal tachycardia up to 130 beats per minute (BPM), fetal tachycardia up to 200 BPM, maternal hypotension with blood pressure (BP) of 84/49 mmHg, and intermittent desaturations to 94% on room air. She continued to endorse subjective fever, malaise, and dyspnea. Her initial laboratory workup revealed leukocytosis of 16.7 × 10^9^/L and thrombocytopenia of 68 × 10^9^/L. Blood cultures eventually revealed *Streptococcus agalactiae* (GBS) bacteremia, suspected due to a urinary source.

A transthoracic echocardiogram (TTE) was performed to evaluate for endocarditis. TTE demonstrated a 1.2 × 1.0 cm vegetation on the aortic valve, specifically on the ventricular aspect of the anatomical left coronary cusp. This cusp was noted to prolapse into the left ventricular outflow tract (LVOT) causing severe eccentric severe aortic insufficiency (AI). In addition, there was mild-to-moderate AS with a peak velocity of 3.1 m/s, a mean gradient of 18 mmHg, and an area of 1.0 cm^2^.

A multidisciplinary team including cardiothoracic surgery, cardiac anesthesiology, and maternal-fetal medicine was convened. The team elected to proceed with emergent cesarean delivery (CS) under general anesthesia to facilitate intraoperative transesophageal echocardiogram (TEE).

**Figure 1:**
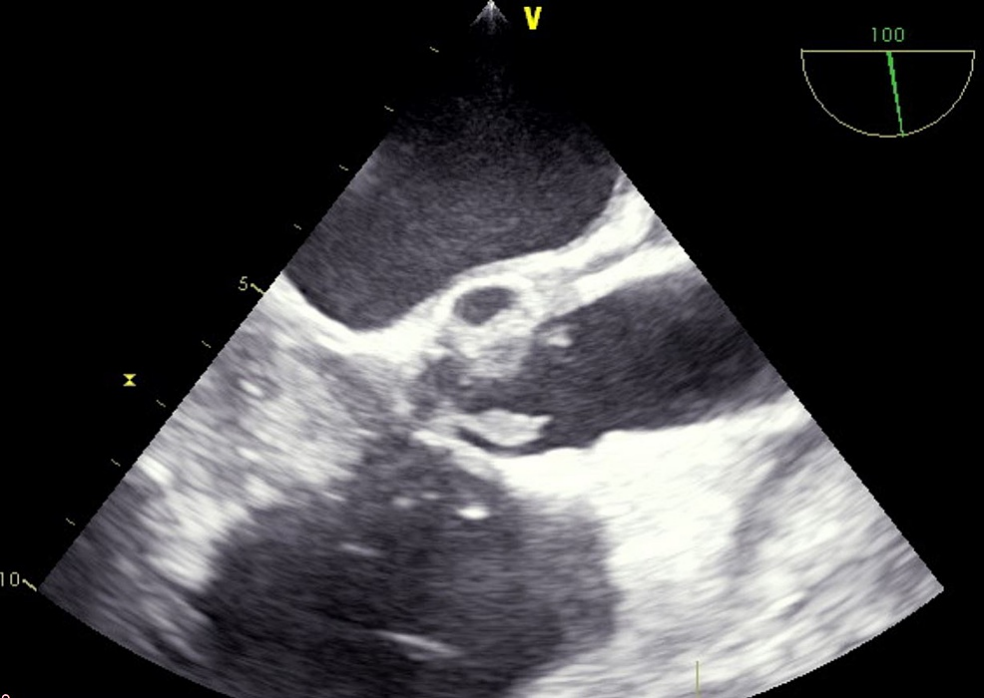
Transesophageal image at the mid-esophageal aortic valve long-axis view demonstrating diffuse bulky vegetations.

**Figure 2:**
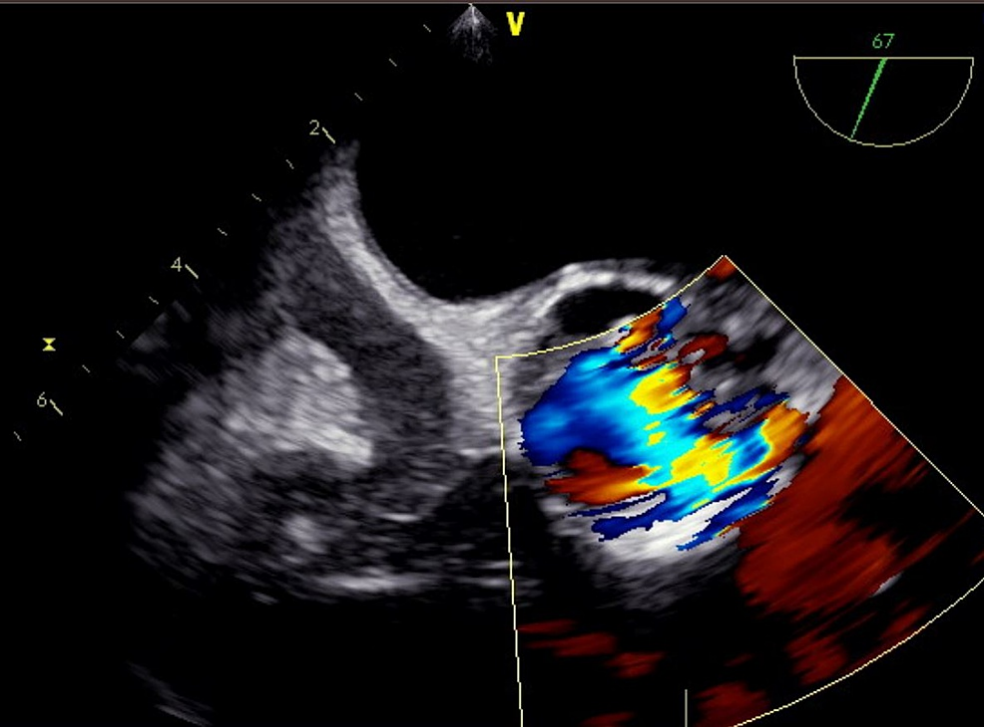
Transesophageal images at the mid-esophageal aortic valve short-axis view with the color Doppler box positioned over the aortic valve demonstrating continuous flow during systole and diastole, indicative of aortic insufficiency.

A preoperative radial arterial line was placed for closer hemodynamic monitoring. Induction was achieved with 0.2 mg/kg of etomidate, 1.3 mg/kg of lidocaine, 1.7 mg/kg of propofol, and 1.5 mg/kg of succinylcholine, followed by easy intubation. The patient remained hemodynamically stable throughout the case, and the delivery was uneventful. Intraoperative TEE demonstrated a bicuspid aortic valve with diffuse, bulky vegetations studding both leaflets protruding into the LVOT, with the largest vegetation measuring 2 cm in length (Figures 1, 2). The leaflets were perforated at two locations, leading to mild AS and severe AI; vena contracta of the two regurgitant jets measured 0.8 cm and 0.4 cm. A small aortic root abscess was also discovered on the mitral side of the aortic valve. The mitral valve did not have vegetations. The patient was extubated without complications.

Two days after delivery, the patient decided to undergo mechanical aortic valve replacement (17-mm St. Jude Medical Hemodynamic Plus valve), aortic annular enlargement, subannular abscess debridement, and aortic root reconstruction with bovine pericardium. Surgical findings included pericardial purulence and large-seeded vegetation on the bicuspid aortic valve. The root abscess tracked into the fibrosa of the heart, posterior aorta-ventricular wall, and the dome of the left atrium requiring extensive debridement. The patient’s chest was left open and packed given concerns of continued bleeding after extensive debridement. She was closed on postoperative day six after multiple washouts. The patient was extubated on postoperative day nine and discharged on postoperative day 25.

## Discussion

The condition

Congenital BAV has an incidence of 1-2% in the general population and is the most common congenital valve malformation [1-3,5,6]. The exact pathogenesis is unknown but is theorized to be due to aberrant aortic cusp development during valvulogenesis. This leads to a “bicuspid” conformation of unequal cusp sizes when two cusps fuse to form one larger leaflet [5-7]. In addition, BAV is frequently associated with aortic pathologies, including root and ascending aortic dilatation, interrupted and hypoplastic arch, coarctation of the aorta, patent ductus arteriosus, and coronary vasculature abnormalities, suggesting shared developmental pathogenesis of the aorta and aortic valve [2,5-8].

In patients afflicted with BAV, there is likely a genetic defect in collagen and elastin metabolism with progressive valvular and vascular complications. The connective tissue properties in these patients are inherently abnormal due to upregulated matrix metalloproteinases and apoptotic smooth muscle cells, drawing clinical parallels to other connective tissue diseases such as Marfan’s and Ehlers-Danlos disease [5,7]. It is this heritable primary aortopathy with chronic inflammation, deterioration, and abnormal remodeling of the aortic media that lend to an increased propensity for aortic dilatation, dissection, and aneurysm in 30-50% of BAV patients [4,5,7,8].

Valvular complications associated with BAV include AS, AI, and infective endocarditis. Stenosis occurs from premature fibrosis and accelerated calcification of the abnormal leaflets, regurgitation from prolapse, fibrotic retraction of the aberrant cusps, and annular dilation [5-8]. Valvular complications often worsen in pregnancy, leading to heart failure, arrhythmias, and myocardial infarction [4].

Infective endocarditis typically involves anatomically abnormal cardiac valves and occurs in 1:8,000 of all pregnancies [9-11]. In BAV, susceptible endothelium allows platelets and fibrin thrombi to form, creating a nidus for seeding and proliferation of microbial organisms [9]. Vegetations not only lead to AS/AI their dislodgement can cause acute vascular insufficiencies such as myocardial, pulmonary infarctions, limb ischemia, or strokes [11,12]. Surgery is indicated if severe and progressive symptoms, heart failure, embolic events, or abscess leading to valve dehiscence are present [12].

Physiologic changes of pregnancy and its impact on bicuspid aortic valve management

Pregnancy causes profound physiologic changes that compound pre-existing hemodynamic risk factors in patients with BAV. Cardiac output (CO) progressively increases throughout pregnancy and peaks at the 36th week of gestation, reaching 130-150% of pre-pregnancy capacity [2,10,13]. This hyperdynamic state subsequently leads to increased vascular wall tension, imposing additional shearing force on the aorta [1,13].

In addition, hormonal changes also cause weakening in the aortic wall. Estrogen and progesterone induce histological changes similar to that cystic medial necrosis in the tunica media and intima [1,4,14]. Thus, pregnancy has historically been associated with an increased risk of aortic dissection, which is especially pronounced in those with an aortopathy [1,3].

Medical and surgical management of bicuspid aortic valve

Parturient patients with BAV can experience safe pregnancies [3]. The key factor for risk stratification is the aortic diameter, with ≤4.5 cm indicating lower risk. Stringent hemodynamic regulation with beta-blockade and serial echographic monitoring is advised to rule out further cardiac dysfunction and progression in aortic diameter [3,4,14].

Approximately one-third of BAV patients ultimately undergo surgery involving the valve, aorta, or both [3]. AS or AI leading to critical narrowing, heart failure, or severe clinical symptoms are common surgical indications [2,15]. Concomitant replacement of the ascending aorta should be performed if the diameter exceeds 4.5 cm [2,16]. The aortic root can be spared if no dilatation is present, but prophylactic surgery before pregnancy should be considered if the diameter exceeds ≥5.0 cm [2,13,14].

Anesthetic management of bicuspid aortic valve during pregnancy

Pregnancy in BAV patients is typically well-tolerated barring severe AS or AI leading to heart failure or significant aortic dilation exceeding ≥4.5 cm. Similar to patients without BAV, controlled vaginal delivery with epidural anesthesia and instrument assistance during the second stage is the preferred mode of delivery due to decreased risk of hemodynamic shifts with vaginal deliveries [2,4,14]. Neuraxial anesthesia can also modulate the maternal pain response during parturition; the sympathectomy can lead to decreased systemic vascular resistance and decreased venous return and preload. Its effects can assist in minimizing aortic wall stress and the effect of CO augmentation during contractions [4]. General anesthesia and CS are considered in severe valvulopathy, aortic disease with a high risk of rupture, or dissection to avoid increased CO in the context of uterine contractions [4]. BP and heart rate (HR) must be carefully monitored, especially during the peripartum period, given that the risk of aortic dissection is the highest late during gestation and shortly after delivery [2].

Given our patient’s profound thrombocytopenia, the risk of epidural hematoma outweighed the benefits of the neuraxial technique. We elected to perform general anesthesia for the CS to facilitate the use of intraoperative TEE. HR was maintained above 80 BPM to reduce the regurgitant fraction and BP was carefully titrated to reduce shear stress on the aorta while ensuring adequate uterine blood flow for fetal perfusion. Although preload is critical in patients with AS, we chose to be judicious with our fluid resuscitation, given the hypervolemic state associated with pregnancy. As the patient remained hemodynamically stable throughout the CS and TEE did not indicate worsening heart failure, we elected to extubate the patient with admission to the intermediate care unit before her cardiac surgery two days later.

## Conclusions

BAV is a complex vascular disease with an abnormality of connective tissue, specifically implicating the aortic valve and the ascending aorta. BAV is also rarely a benign disease because given time every individual with a BAV will develop AS and/or AI due to the anatomical deviation of the leaflets. It often causes significant morbidity and mortality in the peripartum population. Physiologic blood volume, HR, stroke volume, and aortic root size are all increased in pregnancy which further exacerbate the underlying condition. Clinical management is fundamentally challenging and must be individualized. Given thoughtful medical, surgical, and anesthetic management of pregnant patients with BAV, they can often experience safe pregnancies.
